# Tuina for periarthritis of shoulder: A systematic review protocol

**DOI:** 10.1097/MD.0000000000019332

**Published:** 2020-03-13

**Authors:** Jian Ai, Youkang Dong, Qidong Tian, Chunlin Wang, Ming Fang

**Affiliations:** a Yunnan University of Traditional Chinese Medicine First Affiliated Hospital; b College of Acupuncture-Moxibustion and Tuina, Yunnan University of Traditional Chinese Medicine, Kunming; c Shanghai University of Traditional Chinese medicine, Shanghai, China.

**Keywords:** periarthritis of shoulder, protocol, systematic review, Tuina

## Abstract

**Background:**

Periarthritis of shoulder (PAS) symptom is one of the leading causes prompting many patients to seek treatment. Tuina is a common treatment for PAS in China. But at present, there is no systematic evaluation report on its therapeutic effectiveness and safety. This protocol aims to reveal the efficacy and safety of Tuina for treating PAS.

**Methods:**

The following databases will be searched by electronic methods: PubMed, EBASE, WHO International Clinical Trials Registry Platform, Embase, the Chinese Biomedical Literature Database (CBM), Wan-fang Data (WANFANG), the China National Knowledge Infrastructure (CNKI), and other sources from inception to December 2019. Bias risk, subgroup analysis, data synthesis, and meta-analyses will be assessed with RevMan V.5.3 software if the data is met inclusion conditions.

**Results:**

This study will present a quality evidence of Tuina for the treatment of PAS patients.

**Conclusion:**

The systematic review will present reliable evidence to judge whether or not Tuina is a safe and effective intervention for PAS patients.

**PROSPERO registration number:**

CRD42019147445.

## 1 Introduction

The primary symptoms of shoulder periarthritis are pain in different part of the joint and decreased range of motion. The self-reported prevalence is 16% to 26%.^[1,2]^ Shoulder pain is the cause of a lot of sick leave in western countries,^[3,4]^ about 13% of patients take sick leave each year for shoulder pain, at a cost of about $7 billion in the United States, and 26% of occupational patients have shoulder pain in French.^[5–7]^ The incidence is 8% in urban population, and women over 40 are more likely to develop the disease, the incidence is 45% in China.^[8]^ Shoulder periarthritis in traditional Chinese medicine (TCM) known as frozen shoulder and adhesive capsulitis due to the limited activity of the shoulder joint, and it has an impact on people's daily activities and work.^[9]^ Shoulder pain and limited mobility affect people in every way, prompting treatment for this disease is essential.

The treatment of shoulder pain mainly includes restoration of shoulder joint function and reduction of pain intensity.^[10]^ There are many related conservative treatment forms for the treatment of chronic shoulder pain, including corticosteroids, non-steroidal anti-inflammatory drugs and other physical therapy, and the effectiveness of most treatment methods has not been confirmed.^[11]^ Tuina is the use of a certain part of the hand or limb by a doctor to act on a certain part of the patient to press, push, grasp, roll, pinch, etc, which could produce biological effect, improve the corresponding clinical symptoms.^[12]^ Studies have shown that Tuina can increase the concentration of Ca2^+^ in immune system mast cells,^[13,14]^ it can enhance immune function, unblocking and collateral meridians, activate Qi and blood, and improve the flow of Qi.^[15,16]^ It has been widely used in China and many other countries. However, at present, there is no systematic evaluation report on the efficacy and safety of Tuina in the treatment of shoulder periarthritis. The purpose of this paper is to systematically review and analyze the relevant literature, provide effective and safe evidence.

## 2 Methods

The registration number for this system evaluation is (CRD42019147445), which was registered in PROSPERO international prospective register of systematic review.

### 2.1 Selection criteria

#### 2.1.1 Types of studies

The systematic review will include randomized controlled clinical trials (RCTs) and quasi-randomized controlled clinical trials (quasi-RCTs) of PAS comparing forms of Tuina with/without additional treatment against placebo or no treatment or sham or same additional treatment. Designs such as case reports, animal experiment, and retrospective studies will be rejected.

#### 2.1.2 Types of patients

Patients diagnosed with periarthritis of the shoulder regardless of sex, age, race, or severity and duration of disease. Studies comparing two different types of Tuina or surgical procedures will be excluded.

#### 2.1.3 Types of interventions and comparisons

The studies of Tuina as the only experimental intervention. It also includes other similar Tuina interventions such as massage, manipulation, Chinese medicine massage, Chinese massage, etc. Trials that evaluate Tuina as a combination to other therapies will also be included.We will include multiple control interventions as well: drug therapy, behavioral therapy, physiotherapy. and no treatment, placebo or acupuncture will also be included.

#### 2.1.4 Types of outcomes

PAS symptom will be measured with the Visual Analog Scale (VAS) (0–10), the ability assessment of daily living activities (ADL). The secondary outcome will include adverse events incidence and shoulder range of motion (ROM).

### 2.2 Search methods for identification of studies

#### 2.2.1 Electronic searches

The following Electronic databases will be searched without language restriction from the inceptions to December 2019: PubMed, the Cochrane Library, web of science, EBASE, ICTRP, Embase, the China National Knowledge Infrastructure (CNKI), and Chinese Biomedical Literature Database (CBM), Wan-fang Data (WANFANG), Chinese Science and Technology Periodical Database (VIP). The search strategy will be based on guidance provided by the Cochrane Manual, including the following medical subject headings grid terms and variants:“stiff shoulder,” “shoulder periarthritis,” “frozen shoulder,”“shoulder pain,”“shoulder cupsulitis,” “Shoulder osteoarthritis,”“Shoulder osteodystrophy,”“Tuina,”“Chinese Tuina,”“massage,” “massge therapy,”“Chinese massage,” “Manipulation,” “Chinese manipulative therapy,” “Chinese manipulation,” and all possible spellings of “Periarthritis of shoulder” and “Tuina” (Table 1). All search terms are included in this table, and other searches will be based on these results.

**Table 1:**
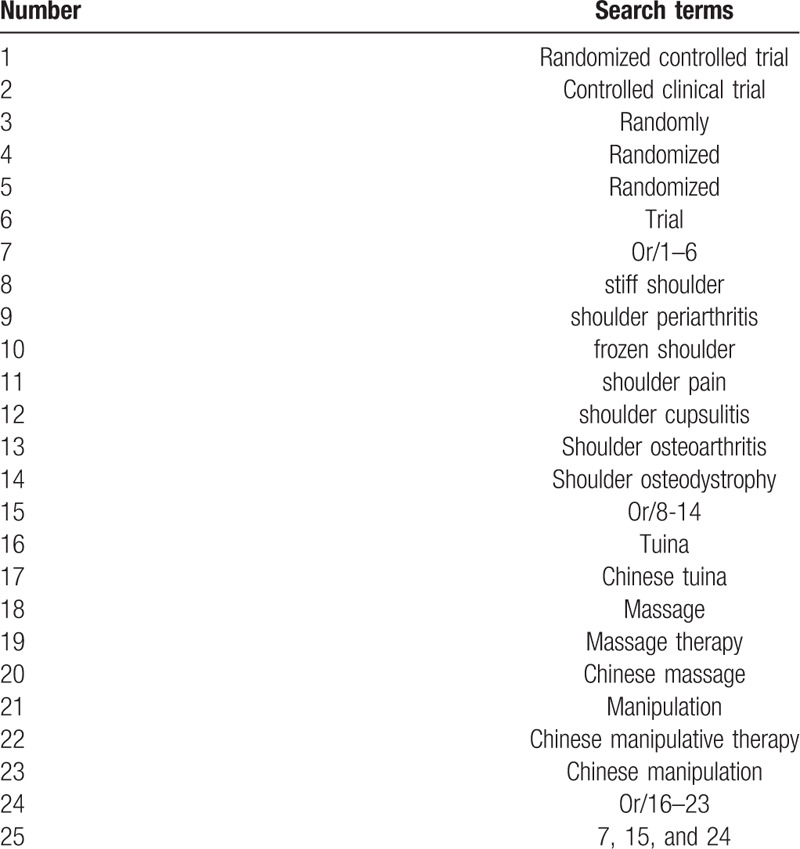
Web of science search strategy.

### 2.3 Data collection and analysis

#### 2.3.1 Selection of studies

**Figure 1:**
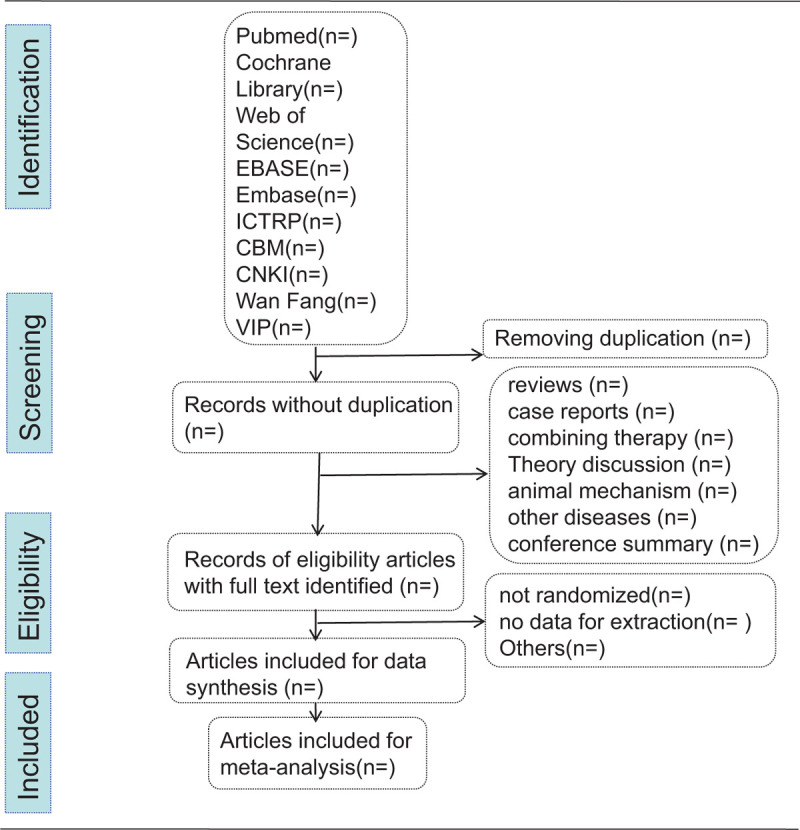
Flow diagram of studies identified.

Jian Ai and Qidong Tian will discuss, and determine research selection process according to the above criteria. We will check the qualifications of titles and summaries of the search study, and evaluate the texts of the remaining studies then include them, after getting the copy from the management system. If there is difference in the research choices, we will resolve it through consulting Min Fang. Researchers Chunlin Wang, Youkang Dong will independently separate the results from every survey according to the standardized data. We will extract basic study information such as basic participant information, control measures, interventions, and outcome indicators. If there are different views about extraction process, it will be discussed or negotiated through consulting Min Fang. Any missing information included in the research will be obtained through contacting the concern author. Screening study flow diagram is summarized as Figure 1.

#### 2.3.2 Assessment and quality of included studies

Two independent reviewers Jian Ai, Youkang Dong will separately survey methodological quality with the Cochrane risk of bias tool. The conflicts cannot be settled in the review will search consensus for a third author (Min Fang) as required. Domains need to be evaluated will include: sequence generation; allocation concealment; blinding of participants; blinding of outcome assessment; incomplete outcome data. The bias risk for every item will be classed as “low risk of bias”, “high risk of bias” or “unclear risk of bias”.

#### 2.3.3 Data extraction

Jian Ai and Chunlin Wang will extract data independently from each study. The extracted data contain lead authors, place of study, year of publication, baseline participant characteristics, intervention, sample size, intervention duration, randomized methods, and distributional concealment, tracking, blinding methods, dropping out and exit, adverse event, outcome measures. For the ambiguity in the studies, it will be solved by expert discussion. We will resolve any opinion differences by reviewing the original document and expert discussion.

#### 2.3.4 Measures of treatment effect

Risk ratio (RR) with 95% confidence interval (95% CIs index) will be used for dichotomy data. Standard mean difference (SMD) or standard (MD) with 95% confidence interval will present continuous results. Other binary data will be changed into RR form.

#### 2.3.5 Dealing with missing data

In view of the lack of data to be extracted from the data contained, if there is incomplete data, we will contact the author by telephone or email. If missing data is not available, if necessary, we will use replacement values to enter the missing data, or through consultation with experts.

#### 2.3.6 Assessment of heterogeneity

The research will be performed with Review Manager version 5.3 software. *P* < .05 will be defined as statistically significant between studies. Heterogeneity will be evaluated by I^2^ statistic. A random effects model will be used if the heterogeneity statistical results is significant (an I^2^ value less than 50%), If not, we will use a fixed effects model, and use Standardized mean differences (SMDs), the corresponding 95% confidence interval (CIs) for further data.

#### 2.3.7 Assessment of reporting bias

The report deviation will be determined according to the funnel diagram and the support obtained from statistical testing.

#### 2.3.8 Data synthesis

We will consider whether to conduct meta-analysis according to clinical research. Clinical research includes the research design of intervention method, measurement method, treatment time and whether the control group chooses the same or not. When a couple of good multiple homogeneity studies are included, we will conduct a meta-analysis with Review Manager 5.3.5. If I^2^ < 50%, fixed effect model and random effect model I^2^ > 50% were selected. Otherwise, we will not be able to implement the meta-analysis.

#### 2.3.9 Subgroup analysis

If the above clinical trial Causes Heterogeneity, we will perform a subgroup analysis based on interventions, different controls, treatment duration, and outcome measurements. We’ll tabulate the adverse reactions and then make an evaluation.

#### 2.3.10 Sensitivity analysis

We will repeat the system evaluation with different types of study, make multiple tests to test the robustness of the review process decisions, which mainly includes the quality of the method, the sample size and the selection of missing data, and we will observe the fluctuation of the results.

## 3 Discussion

Shoulder pain is one source of distress. This disease has brought economic, physical and psychological burdens to many people in all countries and brought great suffering. It has aroused more and more concern all over the world.^[17–20]^ Tuina is a common therapeutic method for PAS in China, it has a long history as an effective natural therapy without side effects,^[21]^ But at the present there is no systematic evaluation report on its therapeutic effectiveness and safety for the treatment of PAS.

Due to the limited data collected, for example, some documents are still in the private sector and have not been published in the electronic database, this paper may have some limitations. This paper may be the first systematic review of the whole literature on the treatment of scapulohumeral periarthritis with Tuina. The aim is to make an objective, true and high quality evaluation of the existing literature on clinical trials of Tuina for PAS at home and abroad. It will provide evidence for relevant research and clinical application with the EBM method.

## Author contributions

Jian Ai is responsible for part work of the selection of studies, data extraction, assessment and quality of included studies. Youkang Dong take part in part work of the study selection, assessment and quality of included studies. Qidong Tian take part in some work of the selection of studies. Chunlin Wang is responsible for part work of the selection of studies and data extraction. Min Fang is responsible for the design and guidance.
